# Artificial intelligence vs. evolving super-complex tumor intelligence: critical viewpoints

**DOI:** 10.3389/frai.2023.1220744

**Published:** 2023-07-24

**Authors:** Nilesh Kumar Sharma, Sachin C. Sarode

**Affiliations:** ^1^Cancer and Translational Research Lab, Dr. D.Y. Patil Biotechnology & Bioinformatics Institute, Dr. D.Y. Patil Vidyapeeth, Pimpri, Pune, Maharashtra, India; ^2^Department of Oral Pathology and Microbiology, Dr. D. Y. Patil Dental College and Hospital, Dr. D.Y. Patil Vidyapeeth, Pimpri, Pune, Maharashtra, India

**Keywords:** artificial intelligence, evolution, neoplasm, tumor intelligence, tumor heterogeneity, cancer management, prognosis

## Abstract

Recent developments in various domains have led to a growing interest in the potential of artificial intelligence to enhance our lives and environments. In particular, the application of artificial intelligence in the management of complex human diseases, such as cancer, has garnered significant attention. The evolution of artificial intelligence is thought to be influenced by multiple factors, including human intervention and environmental factors. Similarly, tumors, being heterogeneous and complex diseases, continue to evolve due to changes in the physical, chemical, and biological environment. Additionally, the concept of cellular intelligence within biological systems has been recognized as a potential attribute of biological entities. Therefore, it is plausible that the tumor intelligence present in cancer cells of affected individuals could undergo super-evolution due to changes in the pro-tumor environment. Thus, a comparative analysis of the evolution of artificial intelligence and super-complex tumor intelligence could yield valuable insights to develop better artificial intelligence-based tools for cancer management.

## Tumor evolution

Tumor evolution refers to the changes that occur in a cancerous tumor over time as it grows and spreads (Hanahan and Weinberg, 2011; Lyssiotis and Kimmelman, 2017). These changes are the result of genetic mutations and changes in gene expression that can give rise to new subpopulations of cells within the tumor (Lyssiotis and Kimmelman, 2017; Balaparya and De, 2018). Over time, these subpopulations may accumulate subsequent mutations that confer enhanced survival and heightened proliferative capacity, thereby culminating in the emergence of a more formidable tumor exhibiting either heightened aggressiveness or treatment resistance (Balaparya and De, 2018; Gui and Bivona, 2022; Shin and Cho, 2023). Tumor evolution can have important implications for cancer diagnosis and treatment. For example, different subpopulations of cells within a tumor may have different sensitivity to certain types of chemotherapy or targeted therapies, meaning that a single treatment approach may not be effective against all parts of the tumor (Aguadé-Gorgorió et al., 2023; Jiménez-Marín et al., 2023). Additionally, tumors that have undergone significant evolution may be more likely to metastasize or spread to other parts of the body, making them more difficult to treat (Aguadé-Gorgorió et al., 2023; Jiménez-Marín et al., 2023).

Understanding tumor evolution is a major area of research in cancer biology, intending to develop more effective diagnostic and treatment approaches that take into account the complex genetic and cellular changes that occur in cancer over time. Cutting-edge methodologies, such as genomic sequencing and single-cell analysis, now empower researchers to scrutinize the genetic and molecular alterations transpiring throughout tumor evolution with heightened precision, fostering the aspiration of devising more bespoke and efficacious cancer therapies. However, perspectives on the evolution of artificial intelligence and super-complex tumor intelligence are lacking.

## Tumor super-complexity

Tumor super-complexity could be interpreted as referring to the complex and heterogeneous nature of tumors, which can make them difficult to diagnose and treat (Ford, 2017; Gourmet and Walker-Samuel, 2023; Weeden et al., 2023). Cancerous tumors can be composed of many different subpopulations of cells, each with its own genetic and molecular characteristics (Ford, 2017; Gourmet and Walker-Samuel, 2023). These subpopulations can interact with each other in complex ways, creating a dynamic and constantly evolving environment within the tumor. Additionally, tumors can show complex interplays with the neighboring microenvironment, encompassing interactions with the immune system and proximal vasculature, thereby further amplifying their heterogeneity and complexities (Hanahan and Weinberg, 2011; Ford, 2017; Lyssiotis and Kimmelman, 2017; Balaparya and De, 2018; Gui and Bivona, 2022; Aguadé-Gorgorió et al., 2023; Gourmet and Walker-Samuel, 2023; Jiménez-Marín et al., 2023; Shin and Cho, 2023; Weeden et al., 2023).

This complexity can make it difficult to develop effective cancer treatments, as a single approach may not be effective against all of the different subpopulations of cells within the tumor. Additionally, tumors can evolve, developing new subpopulations of cells that may be resistant to previously effective treatments.

To address this complexity, researchers are developing more personalized approaches to cancer treatment, taking into account the genetic and molecular characteristics of the tumor and the unique characteristics of the patient. This may include approaches such as targeted therapies, immunotherapies, and combination therapies that aim to target multiple aspects of the tumor at once. Additionally, advances in technologies, such as genomic sequencing and single-cell analysis, are allowing researchers to better understand the complex nature of tumors and develop more effective and personalized treatments.

## Various forms of intelligence

Intelligence can be defined in several ways including abilities of creativity, reasoning, planning abstraction, logic, understanding, self-awareness, learning, emotional knowledge, critical thinking, and problem-solving (Schwabe and Jobin, 2013; Tang and Marshall, 2018; Boyce et al., 2020). Another view on the meaning of intelligence could be derived from two words 'intelli' and “gence.” The simple meaning of intelli is computing which is a programming environment that helps in the coding process by reducing typos/mistakes and enhancing coding outcomes. On the other hand, “gence” is defined as an entity. Hence, the relevance of intelligence is a programming environment that helps in the coding process by reducing typos/mistakes and enhancing the coding outcomes of an entity. Therefore, the concept of intelligence extended from artificial intelligence to cellular intelligence/biological intelligence and specific contexts of tumor intelligence appears logical and reasonable. In the same context, emergence and views on tumor intelligence and microbial intelligence are well aligned which could explain the molecular programming and reprogramming of DNA, RNA, proteins, metabolites, and various other chemical codes.

## Tumor intelligence

In the context of biological systems including the human body, “cellular intelligence” might refer to the ability of trillions of human cells to communicate with each other, respond to environmental cues, and carry out complex functions such as proliferation, differentiation, self-renewal, and programmed cell death (Schwabe and Jobin, 2013; Tang and Marshall, 2018; Boyce et al., 2020). This type of intelligence may be attributed to various types of cells such as specialized epithelial and stem cells.

In the context of artificial intelligence and machine learning, “cellular intelligence” might also refer to algorithms or models that are inspired by the behavior of cells in biological systems (Ford, 2004; Kaiser, 2013; Schwabe and Jobin, 2013; Agarwal et al., 2014; Tang and Marshall, 2018; Boyce et al., 2020; Yang et al., 2020; Sepich-Poore et al., 2021; Zhou et al., 2022). However, this study refers to the cellular intelligence of trillions of biological cells that assist tissues, organs, and the human body as a system to function and process information in sync with the environment and generate outputs such as growth, proliferation, critical thinking, and creativity. In a more general sense, “cellular intelligence” might refer to any type of distributed, decentralized system in which individual units (whether they are cells, sensors, or computational nodes) work together to achieve a larger goal.

By extending the relevance of cellular intelligence to cancer cells, cancer cell intelligence is not a standard scientific term, and it is not typically and often used in the context of cancer research or treatment. However, cancer cells exhibit certain behaviors that could be seen as intelligent or adaptive and essentially perceived as molecular intelligence that allow them to have advantages and are referred to as tumor hallmarks such as growing and dividing uncontrollably, evading the body's immune system, resisting treatment with chemotherapy or radiation, and hiding from the signals of programmed cell death (Hanahan and Weinberg, 2011; Ford, 2017; Lyssiotis and Kimmelman, 2017; Balaparya and De, 2018; Gui and Bivona, 2022; Aguadé-Gorgorió et al., 2023; Gourmet and Walker-Samuel, 2023; Jiménez-Marín et al., 2023; Shin and Cho, 2023; Weeden et al., 2023). The context of cancer cell intelligence can be thought of with their abilities to communicate with nearby cells to support their growth and survival and can even metastasize or spread to other parts of the body.

Interestingly, the result of genetic mutations and changes in gene expression that allow cancer cells to behave in ways that are advantageous for their survival and growth by achieving certain forms of tumor intelligence cannot be discarded. Since cancer cells can also evolve, adapting to changes in their environment and developing resistance to treatments and adaptive evolving cellular intelligence of tumors may be considered. However, cancer cell intelligence should be misunderstood in the context of routine understanding regarding intelligence as the result of conscious decision-making or planning on the part of the cancer cells. However, it should be thought in a way that lack of conscious decision-making or planning on the part of the cancer cells may be the hallmark of cancer cells as cancer cell intelligence is different from the routine cellular intelligence of normal cells.

Recent research suggests that tumor cells may “precondition” lymph nodes, essentially preparing them for future metastasis (Ford, 2017; Aguadé-Gorgorió et al., 2023; Gourmet and Walker-Samuel, 2023; Jiménez-Marín et al., 2023; Shin and Cho, 2023; Weeden et al., 2023). This preconditioning can involve changes to the lymph node's structure and immune environment, as well as the release of signaling molecules that promote cancer cell survival and growth. This super intelligence impact of tumor cells on distance sites can be analogous to “Cloud Computing.” Understanding the mechanisms behind lymph node preconditioning could lead to new strategies for preventing or treating cancer metastasis.

While cancer cells do exhibit certain adaptive behaviors, there is a significant gap that could resolve that abnormal attributes of tumors are a result of intelligence or consciousness on the part of the cells or vice versa (Hanahan and Weinberg, 2011; Lyssiotis and Kimmelman, 2017; Balaparya and De, 2018). It is well acknowledged that cancer is a super-complex disease that is still not fully understood. Therefore, understanding tumor intelligence may create platforms in the future so that the adaptability of cancer cells in response to growing forms of therapies and environment can be decoded.

## Gut microbial intelligence

The super-complex nature of the tumor is linked with the gut microbiome through various chemical signaling pathways (Ford, 2004; Sepich-Poore et al., 2021; Zhou et al., 2022). In the context of evolving nature of tumor intelligence, it would be reasonable to understand the complexity of gut microbiomes in terms of their abilities to sense the gut environment locally and distant tissues through various chemical messengers. The gut microbiome is suggested to perform complex tasks such as cellular communication with heterogeneous cell types and extracellular communication as a response to external factors such as drugs (Bezdek, 1994; Kaiser, 2013; Agarwal et al., 2014; Yang et al., 2020). In the context of the tumor, the gut microbiome uses chemical signals to communicate that can influence tissue metabolism, which may be one of the critical factors in the initiation and progression of cancer. Therefore, the term gut microbial intelligence is denoted that refers to the abilities of the gut microbiome to perform various cellular and extracellular processes.

Overall, normal gut microbial intelligence in healthy individuals reflects the balanced role of the gut microbiome within the body. Nonetheless, the perturbation of gut microbial intelligence can arise due to external factors, including pharmaceuticals, antibiotics, and synthetic compounds, which can lead to the formation of a dysbiotic microbiome (Bezdek, 1994; Ford, 2004; Kaiser, 2013; Agarwal et al., 2014; Yang et al., 2020; Sepich-Poore et al., 2021; Zhou et al., 2022). Such dysbiotic microbiomes may influence the predisposition to cancer. An interesting proposition may arise to test that various classes of drugs including antibiotics may reduce the population of the gut microbiome and eventually alterations in the quorum sensing by activation or deactivation of genes that may contribute to the pro-tumor microenvironment (Bezdek, 1994; Agarwal et al., 2014; Yang et al., 2020). Therefore, future avenues to understand both normal gut microbial intelligence and pro-tumor gut microbial intelligence may be helpful for the better management of cancer.

## Artificial intelligence

In line with the evolving complexities of tumors with acquired tumor intelligence, artificial intelligence (AI) is being increasingly utilized in cancer research and treatment and has the potential to contribute significantly to our ability to diagnose and treat tumors (Eberhard, 1997; Jiang et al., 2017; Ström et al., 2020; Wilkinson et al., 2020; Bhinder et al., 2021). However, it is important to note that AI is not a magic bullet that can solve all the challenges associated with tumor super-complexity on its own.

One potential application of AI in cancer research is to analyze large amounts of genomic and molecular data from tumors to identify patterns and predict how tumors are likely to evolve (Luchini, 2022; Sarode et al., 2022; You et al., 2022). By analyzing large amounts of data, AI algorithms can identify potential drug targets and develop more effective personalized treatment plans. Additionally, AI can be used to identify potential biomarkers that could be used to diagnose cancer at an earlier stage, improving patient outcomes (Vasan et al., 2019; Forghani, 2023). Generative and progressive AI is proposed to evolve with time and environment which could assist in the right diagnosis and therapies for cancer.

A critical view is that in the future, AI will evolve and learn to predict early detection and new modalities of treatment options including new classes of drugs and inhibitors. A possibility that AI-dictated anti-tumor modalities could lead to adaptations and changes in the tumor with a potential for super-complexities at molecular levels.

However, AI is not a substitute for the insights and expertise of trained medical professionals. While AI algorithms can analyze large amounts of data, they may not always take into account the unique characteristics of each patient with evolving tumor intelligence and super-complexities.

In conclusion, while AI has the potential to be an important tool in the fight against cancer, it is unlikely to be a panacea for the evolving complexity of tumors. However, by combining the strengths of AI with the expertise of medical professionals, we can develop more effective approaches to diagnosing and treating cancer in the future.

## Critical viewpoints

The use of drugs, radiation, and surgical therapies to treat tumors can lead to certain changes within the tumor that can make it more complex or resistant to treatment over time (Ford, 2017; Ward et al., 2021; Aguadé-Gorgorió et al., 2023; Gourmet and Walker-Samuel, 2023; Jiménez-Marín et al., 2023; Weeden et al., 2023; 32). This is because tumors are capable of evolving and adapting in response to treatment, developing new mutations or changes in gene expression that allow them to survive and grow in the presence of treatment. Chemotherapy and radiation therapies are designed to kill rapidly dividing cancer cells, but they may not be effective against certain subpopulations of cells and that can lead to evolution to become more resistant to chemotherapy over time with the accumulation of extra-tumor intelligence.

Hence, a pertinent question is raised on how AI evolution will keep pace with changing tumor intelligence and super-complexities. It is important to note that these therapies remain important tools in the fight against cancer. With careful monitoring and personalized treatment plans, medical professionals can work to minimize the potential negative effects of these therapies and maximize their benefits for patients. Additionally, ongoing research into new treatments and approaches to cancer therapy may lead to the development of more effective and targeted therapies that are better able to counter the evolving complexity of tumors.

One of the conceptual implications of tumor intelligence could be thought of as the possibility of reversal of tumor intelligence that represents various intracellular and extracellular molecular events with certain forms of programming and reprogramming such as genetic, epigenetic, and metabolic adaptations. Therefore, at this juncture, understanding tumor intelligence and avenues to reverse tumor intelligence into normal cellular intelligence may be theoretical but promising in the future. This therapeutic approach can be labeled as “Cancer Intelligence Correction Therapy” or “Cancer Intelligence Modulation Therapy.” Since changing environmental conditions, it would be logical to accept that appreciable human subjects will progress to tumorigenesis and tumor intelligence. Hence, even though theoretical and conceptual, out of box logical and reasonable avenues should be explored.

The evolving paradigm of AI-specific subset of the machine and deep learning helps in diagnosis to therapeutics by mining and predicting molecular events at intracellular and extracellular levels. Interestingly, the predictability of AI depends on the extent of cellular intelligence of cancer cells and is altogether denoted as tumor intelligence. Therefore, the race to survival and adaptations by cancer cells could be modulated by these various therapies and interventions, and this could lead to even further evolution of tumors with acquired intelligence in the form of tumor intelligence.

Theoretically, humans or nature may not be able to stop the evolution of AI and tumor intelligence because we cannot control the adaptive environment and that is beyond the control of humans. Therefore, the race of intelligence between AI and tumor intelligence could be progressive and determining factors for the wellbeing of humans on the earth.

A new perspective has emerged regarding the potential emergence of artificial superintelligence (ASI) in the form of software-based systems, possessing intellectual attributes that surpass those of humans across a broad range of parameters. These parameters could be expanded to include the ability to compete with the increasingly sophisticated intelligence of tumors in the diagnosis and treatment of complex cancers in the future. While ASI remains hypothetical at present, it could represent a natural evolution of AI and be in line with the development of tumor intelligence, ultimately facilitating better management of these diseases.

A pertinent question is impending about the capabilities of AI and machine intelligence to predict not only the occurrence of tumors but also how tumors will evolve and acquire new forms of intelligence in the changing environmental landscape. The future of AI and machine intelligence to aid in the interventional approaches that will program and reprogram cancer cells so that these cells will be guided to normal cellular pathways. A highly intriguing question may arise whether the increase in the tumor intelligence in the case of cancer patients will influence the decrease in the intelligence of other body cells.

In summary, the concomitant evolution of drugs, radiation, and surgical therapies along with the potential emergence of tumor super-complexities and tumor intelligence is predicted. Therefore, the evolution of AI to match tumor super-complexities and tumor intelligence will always be a matter of concern. In the future, better understanding of both the evolution of AI and tumor intelligence could be challenging to human health and society.

## Data availability statement

The original contributions presented in the study are included in the article/supplementary material, further inquiries can be directed to the corresponding author.

## Author contributions

NS: idea/conceptualization, data collection, and manuscript writing. SS: data collection, manuscript writing, and editing.
